# Effects of Different Dietary Starch Levels on Growth and Energy Metabolism of Juvenile Chinese Mitten Crab (*Eriocheir sinensis*) Based on Metabolomic Analysis

**DOI:** 10.1155/anu/2505201

**Published:** 2026-10-01

**Authors:** Yanan Liu, Xiaojie Zhou, Sudong Xia, Zhenyan Cheng, Jinwei Gao, Wenli Zhou, Dan Zhang

**Affiliations:** ^1^ Tianjin Key Lab of Aqua-Ecology and Aquaculture Department of Fishery Science Tianjin Agricultural University Tianjin 300392 China tjac.edu.cn; ^2^ Key Laboratory of Smart Breeding Ministry of Agriculture and Rural Affairs Tianjin Agricultural University (Co-constructed by the Ministry and the Province) Tianjin 300393 China agri.gov.cn

**Keywords:** energy metabolism, *Eriocheir sinensis*, feed starch, growth performance

## Abstract

Starch is a critical energy source in the nutrition of aquatic animals and significantly influences the physiological and metabolic characteristics of the organism. Therefore, determining an appropriate dietary starch level is of great significance for analyzing the nutritional requirements of *Eriocheir sinensis*. This study aimed to evaluate the effects of different dietary starch levels on growth and energy metabolism in *E. sinensis* and to identify an appropriate dietary starch level. A total of 150 healthy *E. sinensis* with an initial weight of 9.49 ± 0.11 g were selected and divided into five groups with different starch levels: 12%, 17%, 22%, 27%, and 32%. Each group consisted of three biological replicates, with 10 experimental crabs per replicate. After the 60‐day feeding trial, samples were collected for the determination of energy metabolism–related enzyme activities and gene expression levels. Targeted metabolomics was employed to measure the content of energy metabolites in the muscle of experimental crabs under different dietary starch levels. The results indicated that the growth performance of *E. sinensis* varied significantly with different dietary starch levels (*p* < 0.05), exhibiting a trend of initially increasing and then decreasing with higher starch levels, with the best growth observed in the group with a starch level of 22%. Regarding energy metabolism, the enzyme activities of hexokinase (HK), pyruvate kinase (PK), and others showed a trend of initially increasing and then decreasing with rising dietary starch levels, and the differences in related gene expression levels were significant (*p* < 0.05). The expression levels of key genes in the adenosine monophosphate (AMP)–activated protein kinase (*AMPK*) and target of rapamycin (*TOR*) pathways also showed significant differences under different starch levels in the feed (*p* < 0.05). Among the 26 energy metabolites obtained by energy metabolomics, 16 metabolites, including flavin mononucleotide (FMN), cyclic AMP (cAMP), and nicotinamide adenine dinucleotide (NAD), reached their highest levels when the starch level in the feed was 22%. The experimental results preliminarily showed that the growth of *E. sinensis* was significantly different under different starch levels in the feed, which was speculated to be related to the active regulation of energy metabolism pathways. When the starch level in the feed was 22%, *E. sinensis* grew better and accumulated more energy metabolites. These findings provide a scientific reference for starch inclusion in diets for *E. sinensis*.

## 1. Introduction

Dietary starch, a polysaccharide carbohydrate, serves not only as a fundamental energy reserve in aquaculture feeds but also as a critical modulator of energy conversion and metabolic processes in aquatic species [1]. An optimal level of dietary starch confers multifaceted benefits in aquatic animal nutrition [2]. Primarily, as a key energy source, starch provides substantial support for healthy growth and sustained energy supply in aquatic organisms [3]. Moreover, its cost‐effectiveness renders starch an economically efficient ingredient in feed formulations, fulfilling dual roles as both a binder and an energy supplement, which aligns well with the economic demands of intensive aquaculture production [4]. However, both insufficient and excessive starch intake can adversely affect aquatic animals [5]. Inadequate starch consumption may lead to energy deficiency and compromised disease resistance, thereby impeding normal physiological development. Conversely, excessive intake can provoke digestive disturbances, disrupt intestinal microbiota homeostasis, and induce metabolic dysregulation, such as fluctuations in blood glucose levels.

In formulated crustacean feeds, dietary starch plays a crucial role in dictating total energy intake and metabolic utilization efficiency, thereby exerting a primary regulatory influence over energy metabolism [6]. A deficiency in dietary starch can induce a metabolic shift toward protein catabolism to compensate for the energy deficit, resulting in metabolically inefficient energy production and subsequent growth impairment [7]. Conversely, starch levels that exceed metabolic capacity can precipitate metabolic overload, adversely affecting physiological homeostasis and growth performance [8]. Consequently, dietary starch content is a pivotal determinant modulating the balance between growth and energy metabolism in crustaceans. The mechanistic basis of this regulation involves two central nutrient‐sensing pathways: the adenosine monophosphate (AMP)–activated protein kinase (AMPK) pathway is activated under conditions of energy deficit, such as low starch availability, promoting catabolic processes to restore energy homeostasis [9]. In contrast, the target of rapamycin (TOR) pathway is stimulated by nutrient sufficiency, exemplified by adequate starch intake, driving anabolic processes, including protein synthesis [10]. Together, these pathways form an integrated regulatory network that precisely coordinates energy metabolism in response to dietary starch availability in crustaceans. In aquatic animals, muscle is a major edible tissue and an important site of energy utilization for movement and growth. Changes in glycolysis, the tricarboxylic acid (TCA) cycle, nucleotide metabolism, and redox‐related cofactors such as nicotinamide adenine dinucleotide (NAD), NAD phosphate (NADP), and flavin mononucleotide (FMN) can reflect shifts in energy homeostasis under different nutritional conditions [5, 11, 12]. Therefore, muscle energy metabolite profiling may help clarify how dietary starch regulates energy allocation and growth in *Eriocheir sinensis*.

The Chinese mitten crab (*E. sinensis*) is a highly economical crustacean with both considerable edible value and remarkable breeding benefits [13]. The growth, development, nutrient metabolism, and quality formation of *E. sinensis* are closely associated with the content of starch‐based additives in formulated feeds [14]. Previous studies have indicated that dietary starch supplementation exerts significant effects on the digestive function and growth rate of *E. sinensis* [15]. In addition, improvements in intestinal health and molting performance are recognized as relevant indicators reflecting the beneficial roles of starch in this species [16]. Taken together, dietary starch first affects feed utilization and digestive capacity, then alters glucose availability and energy production, and finally influences growth‐related physiological processes through energy‐sensing pathways. However, the link between dietary starch level, growth performance, and energy metabolism in juvenile *E. sinensis* remains insufficiently understood. Therefore, this study aims to evaluate the effects of different dietary starch levels on growth performance and energy metabolism in *E. sinensis*.

## 2. Materials and Methods

### 2.1. Source and Management of Experimental Crabs

This experiment was conducted in the aquaculture ponds of the Freshwater Station, Tianjin Institute of Fisheries, with a recirculating aquaculture system (RAS) as the main culture environment. Healthy, active, and disease‐resistant juvenile crabs meeting the experimental standards were selected and acclimated in 35 m^2^ cement tanks with continuous aeration for 2 weeks. A total of 150 juvenile crabs, with an average wet body weight of 9.49 ± 0.11 g, were randomly divided into five dietary groups with three replicates per group and 10 crabs per replicate. Crabs in each replicate were reared in glass tanks (50 cm × 30 cm × 35 cm) connected to a RAS. The feeding trial lasted for 60 days. The flow rate was maintained at ~1.33 L/min per tank. Water temperature was 24–26°C, dissolved oxygen > 6 mg/L, and pH ~7.5. Unionized ammonia nitrogen (NH_3_‐N), total ammonia nitrogen (TAN), and nitrite nitrogen (NO_2_^-^‐N) were maintained below 0.05, 0.20, and 0.10 mg/L, respectively. To reduce aggressive interactions and cannibalism, PVC pipe shelters were placed in each tank, and the crabs were checked daily.

### 2.2. Feed Formulation

As shown in Table 1, the feed formulations in this experiment were prepared by mixing high‐quality protein, premium fish oil, fish meal, vitamins, cellulose, and various nutrients. A single‐factor randomized design was adopted, with dietary starch level as the sole experimental variable. The starch levels were selected with reference to the range previously tested in juvenile *E. sinensis*, with a 5% interval used to cover low, moderate, and high starch supply [16]. Five experimental diets were formulated with starch levels of 12%, 17%, 22%, 27%, and 32%, respectively. All ingredients were thoroughly mixed and ground to 30–200 mesh using a high‐speed pulverizer (DFY‐500, Wenling Linda Machinery Co., Ltd., Zhejiang, China; 25,000 r/min). The mixture was then extruded into feed pellets with a diameter of ~2 mm using a twin‐screw extruder, and the pellets were sieved. Subsequently, the pellets were dried to constant weight at 60°C, crushed, and sieved again to serve as experimental diets for *E. sinensis*.

**Table 1:** Experimental diets with different carbohydrate levels and contents of main nutritional components for *Eriocheir sinensis* (g/100 g).

Ingredients	A	B	C	D	E
Peruvian fish meal	32.1	32.1	32.1	32.1	32.1
Soybean meal	16.0	16.0	16.0	16.0	16.0
Wheat starch	12.0	17.0	22.0	27.0	32.0
Microcrystalline cellulose	20.0	15.0	10.0	5.0	0.0
Casein	6.9	6.9	6.9	6.9	6.9
Wheat gluten	5.0	5.0	5.0	5.0	5.0
Fish oil	2.0	2.0	2.0	2.0	2.0
Vitamin‐mineral premix	2.0	2.0	2.0	2.0	2.0
Binder	1.5	1.5	1.5	1.5	1.5
Calcium dihydrogen phosphate	1.0	1.0	1.0	1.0	1.0
Lecithin	1.0	1.0	1.0	1.0	1.0
Cholesterol	0.5	0.5	0.5	0.5	0.5
Total	100	100	100	100	100
Nutritional ingredient (% dry matter)					—
Moisture	7.8	8.1	8.3	8.0	8.2
Crude protein	35.7	35.8	35.3	35.4	35.7
Crude lipid	5.4	5.5	5.2	5.4	5.3
Crude ash	8.4	8.4	8.7	8.6	8.6

### 2.3. Determination of Enzyme Activities

Individual crabs were anesthetized with an ice–water mixture and placed on an ice tray. Because the hepatopancreas is a major site of carbohydrate catabolism and enzyme synthesis in *E. sinensis*, hepatopancreatic tissue was rapidly dissected and collected into 1.5‐mL centrifuge tubes. Extraction buffer was added at a mass∶volume ratio of 1 g∶9 mL, followed by mechanical homogenization. The homogenate was centrifuged at 10,000 r/min for 10 min at 4°C, and the supernatant was collected. The contents of glycogen (Nanjing Jiancheng, A043‐1‐1), lactic acid (Nanjing Jiancheng, A019‐2‐1), and glucose (Nanjing Jiancheng, F006‐1‐1), as well as the activities of hexokinase (HK, Nanjing Jiancheng, A077‐3‐1), glucose dehydrogenase (GDH, Nanjing Jiancheng, M011), pyruvate kinase (PK, Nanjing Jiancheng, A076‐1‐1), and lactate dehydrogenase (LDH, Nanjing Jiancheng, A020‐1‐2) were determined using corresponding assay kits (Nanjing Jiancheng Bioengineering Institute, Nanjing, China) according to the manufacturer’s instructions. Based on the results of preliminary gradient experiments, three representative gradient groups with starch addition levels of 12%, 22%, and 32% were selected for molecular‐level verification and analysis.

### 2.4. Gene Expression Analysis by Quantitative Real‐Time PCR (qRT‐PCR)

Muscle tissues of *E. sinensis* were used to evaluate growth‐ and energy metabolism–related indicators, whereas hepatopancreas samples were used to assess glucose metabolism–related indicators. Total RNA was isolated from ~50 mg of powdered hepatopancreas or muscle tissue using TRIzol reagent. The quality and quantity of the obtained RNA were assessed by a Nanodrop 2000 spectrophotometer (Thermo Scientific), and the integrity was assayed by 1.2% agarose gel electrophoresis.

Genomic DNA was removed by DNase I treatment with incubation at 37°C for 30 min, and DNase I was inactivated by EDTA treatment with heating at 65°C for 10 min; subsequently, cDNA synthesis was achieved by incubating the reverse transcription reaction system at 42°C for 60 min, and the reaction was finally terminated by heating at 70°C for 5 min.

qRT‐PCR was performed on an Applied Biosystems 7500 RT‐PCR system. The 20‐μL reaction mixture contained 10 μL of SYBR Mix, 0.8 μL of each primer, 2 μL of cDNA (300 ng), and 6.4 μL of H_2_O. After initial denaturation at 95°C for 30 s, PCR was performed for 40 cycles of denaturation at 95°C for 5 s and annealing at 60°C for 34 s. As shown in Table 2, the primers used in the experiment were designed based on the published gene sequences retrieved from NCBI. The *β*‐actin gene was used as the reference gene because its expression has been shown to be stable in different organs or under various treatments of *E. sinensis*. All samples were measured in triplicate, and the 2^−ΔΔCT^ method was used to analyze the qRT‐PCR results. Based on the results of preliminary gradient experiments, three representative gradient groups with starch addition levels of 12%, 22%, and 32% were selected for molecular‐level verification and analysis.

**Table 2:** Primer sequences of energy metabolism related genes in *Eriocheir sinensis*.

Gene	Primer	Access number
*CHH*	F: 5′‐CATCAGTTCTCCTTCTGGTCGC‐3′	KJ660843.1
	R: 5′‐CCTGCCTCTTTCTTCTGACGG‐3′	
*ECR*	F: 5′‐GTCGTTATGACGCAAAGACCAAT‐3′	KF234770.1
	R: 5′‐TGTTTGCCACATACGCACTCA‐3′	
*MIH*	F: 5′‐AGCGGACATTTCTCTCCCAAG‐3′	MH050900.1
	R: 5′‐AACACGGCTGCCACCAAC‐3′	
*S6K1*	F: 5′‐CAAGCATAGAGTCACCATCGGA‐3′	MN177594
	R: 5′‐GACACCATACTGTTAGGGGAGCA‐3′	
*EIF4E*	F: 5′‐TGGAATCAAACCTATGTGGG‐3′	XM_047869202.1
	R: 5′‐GTCCTCCTGGAAGCGTA‐3′	
*RAPTOR*	F: 5′‐AGGTGTCAAGGTTATCAGGAGGAG‐3′	XM_047880321.1
	R: 5′‐CTAACTGTTTTCATTCGTTCCTTCA‐3′	
*TOR*	F: 5′‐TGCCAACGGGTGGTAGA‐3′	XM_047874598.1
	R: 5′‐GGGTGTTTGTGGACGGA‐3′	
*4EBP*	F: 5′‐ATGTCTGCTTCGCCCGTCGCTCGCC‐3′	MW687141.1
	R: 5′‐GGTTCTTGGGTGGGCTCTT‐3′	
*AMPKα*	F: 5′‐ACACTACCATTTGACGATGAGCA‐3′	KP272116.1
	R: 5′‐AGGAGGTGGGAACAGATAAGCA‐3′	
*AMPKβ*	F: 5′‐CAAAACTTATGGTGTTCAGGCTC‐3′	KP272117.1
	R: 5′‐ACGAGATTGGTGTGTCCTTATTTAG‐3′	
*AMPKγ*	F: 5′‐CTCATTCATCATAAGATTCACCGC‐3′	KP272118.1
	R: 5′‐CTGGCTGGCAGTCTGTATGTTAG‐3′	
*SIRT1*	F: 5′‐TCTGGAGGAAGTGACTGTGGAA‐3′	MG321242.1
	R: 5′‐CGAAGAAGGGCTTGGGGTT‐3′	
*V-ATP*	F: 5′‐ATAGCGTTTCTCCCAACTGTGC‐3′	Unigene0038386
	R: 5′‐GCCAAACTGCCCAGCATACATA‐3′	
*LKB1*	F: 5′‐TAGCGAACGGTTATCCCTGAAT‐3′	XM_047870091.1
	R: 5′‐AGGAACTATGCTTATGCTGTGACG‐3′	
*PGC-1α*	F: 5′‐TCACCTCTGTGGATGGAAAAGC‐3′	XM_047865509.1
	R: 5′‐CAGAAAGATGATGTTGGTCCCC‐3′	
*PK*	F: 5′‐GCATTATTCCCATCCACTACACTG‐3′	EF102105.1
	R: 5′‐GAAACCACACTCCTTGCCATAC‐3′	
*HK*	F: 5′‐ATCGGCAAGTTAGATACGCAGG‐3′	EF102106.1
	R: 5′‐GGACACCACGGTAGGAAATGA‐3′	
*GLUT1*	F: 5′‐GAGCAACTTCTTGGGAATAGCA‐3′	KM201335.1
	R: 5′‐GCTTCATCCTCTAACTGGCGTG‐3′	
*β-Actin*	F: 5′‐GCATCCACGAGACCACTTACA‐3′	KM516908
	R: 5′‐CTCCTGCTTGCTGATCCACATC‐3′	

### 2.5. Metabolite Extraction and LC‐MS/MS Analysis

A total of 60 mg of muscle tissue was homogenized in 500 μL of a cold methanol: acetonitrile: H_2_O mixture (2:2:1, v/v/v) using an MP homogenizer at a speed of 6.0 M/s for two intervals of 60 s. The homogenate was sonicated twice for 30 min at 4°C and then centrifuged at 14,000× g for 20 min at 4°C. The resulting supernatants were dried under vacuum. The residues were subsequently reconstituted in 100 μL of a 1:1 (v/v) acetonitrile: water solution and centrifuged at 14,000 g for 15 min at 4°C.

LC–MS/MS analysis was conducted using an Agilent 1290 Infinity ultrahigh‐performance liquid chromatography system. The mobile phase consisted of 10 mM ammonium acetate in water (A) and acetonitrile (B), with the following elution gradient: at 0 min, 10% A and 90% B; at 18.0 min, 60% A and 40% B; at 18.1 min, 10% A and 90% B; at 18.2 min, 15% A and 85% B; and at 23.0 min, 15% A and 85% B, delivered at a flow rate of 400 µL/min. The injection volume was 4 µL. Primary and secondary MS data in MS mode were collected using Cluster 3.0 and Java Tree View software. The parameters for the ESI ion source were as follows: source temperature of 450°C, ion source gas 1 (Gas 1) at 45, ion source gas 2 (Gas 2) at 45, curtain gas (CUR) at 30, and ion spray voltage floating (ISVF) at −4500 V. MRM mode was employed for detection of the ion pairs.

### 2.6. Data Calculation and Analysis

The overall workflow of this experiment is shown in Figure 1. Growth performance parameters were calculated as follows:

**Figure 1:**
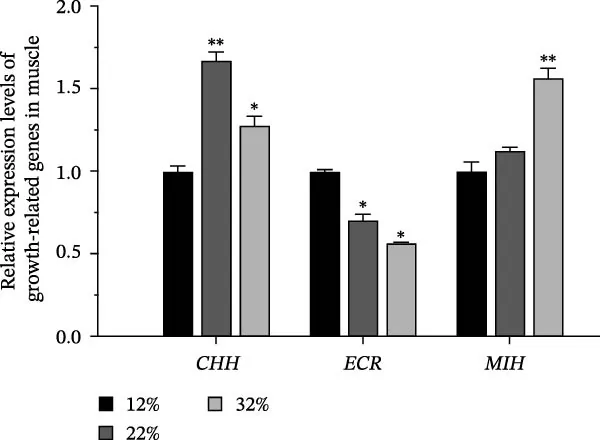
Effects of different dietary starch levels on the relative expression levels of growth‐related genes in *Eriocheir sinensis*.  ^∗^ indicates *p* < 0.05, and  ^∗∗^ indicates *p* < 0.01.

SR%=NfNi×100,

SGR% day−1=lnWf−lnWit×100,

TWGR%=Wf−WiWi×100,

DWGR% day−1=Wf−WiWi×t×100,

FCR=feed intakeweight gain.

*W*_*i*_ and *W*_*f*_ represent the initial and final average body weights of crabs, respectively; *N*_*i*_ and *N*_*f*_ represent the initial and final numbers of crabs, respectively; and *t* represents the feeding duration in days. Data are presented as mean ± SE. Normality and variance homogeneity were assessed using Shapiro–Wilk and Levene’s tests, respectively. Data with homogeneous variances were analyzed by one‐way ANOVA followed by Duncan’s test, whereas data with heterogeneous variances were analyzed by Welch’s ANOVA followed by the Games–Howell test (*p* < 0.05). Peak areas and retention times of chromatograms were acquired using Multiquant software. Retention times were calibrated with metabolite standards for metabolite identification [17].

## 3. Results

### 3.1. Effects of Different Dietary Starch Levels on Growth Performance of *E. sinensis*

As shown in Table 3, different dietary starch levels had significant effects on the final body weight, carapace height, carapace length, carapace width, daily weight gain rate (DWGR), specific growth rate (SGR), and total weight gain rate (TWGR, 60 days) of *E. sinensis* at the end of the experiment (*p* < 0.05), with an overall trend of first increasing and then decreasing. Among all groups, the group with 22% dietary starch showed significantly higher growth performance indices than the 12% and 32% starch groups (*p* < 0.05). *E. sinensis* exhibited optimal DWGR and SGR when dietary starch levels ranged from 17% to 22%. The feed conversion ratio (FCR) showed a trend of first decreasing and then increasing with the increase in dietary starch content. A lower FCR reduces feed costs while enhancing the economic value of the feed, which is beneficial to the growth of *E. sinensis*.

**Table 3:** Growth performance of juvenile *Eriocheir sinensis* fed diets containing different starch levels.

Different dietary starch levels	12%	17%	22%	27%	32%
Initial average body weight	9.33 ± 0.12	9.60 ± 0.15	9.40 ± 0.06	9.67 ± 0.03	9.43 ± 0.13
Final average body weight	18.50 ± 1.00^b^	22.46 ± 1.81^ab^	23.48 ± 1.92^a^	20.71 ± 1.04^ab^	19.31 ± 0.15^ab^
Carapace height	16.70 ± 0.21^ab^	17.27 ± 0.40^ab^	17.99 ± 0.45^a^	17.00 ± 0.08^ab^	16.41 ± 0.58^ab^
Carapace width	32.94 ± 0.28^b^	34.11 ± 0.51^b^	35.56 ± 0.42^a^	33.64 ± 0.12^b^	33.84 ± 0.37^b^
Carapace length	31.18 ± 018^b^	32.11 ± 0.59^b^	33.62 ± 0.46^a^	32.14 ± 0.13^b^	31.83 ± 0.36^b^
Specific growth rate (SGR)	1.12 ± 0.07^b^	1.41 ± 0.14^ab^	1.51 ± 0.14^a^	1.27 ± 0.08^ab^	1.19 ± 0.02^ab^
Total weight gain rate (TWGR, 60 days)	98.03 ± 8.54^b^	134.01 ± 18.80^ab^	152.09 ± 19.17^a^	114.20 ± 10.40^ab^	104.75 ± 2.37^b^
Daily weight gain rate (DWGR)	1.63 ± 0.14^b^	2.23 ± 0.31^ab^	2.53 ± 0.32^a^	1.90 ± 0.17^ab^	1.75 ± 0.04^b^
Feed conversion ratio (FCR)	2.70 ± 0.42	2.42 ± 0.57	2.04 ± 0.13	2.56 ± 0.07	2.60 ± 0.13
Survival rate (SR)	96.67 ± 3.33	96.67 ± 3.33	93.33 ± 3.33	96.67 ± 3.33	93.33 ± 3.33

*Note:* Different superscript letters within the same row indicate significant differences among dietary treatments (*p* < 0.05).

Within the scope of this experiment, Figure 1 shows that different dietary starch addition levels resulted in significant differences in the expression of the crustacean hyperglycemic hormone (*CHH*) gene in the muscle of *E. sinensis* among the three groups (*p* < 0.05), showing an overall trend of first increasing and then decreasing. The 22% starch group had the highest gene expression level, while the 12% starch group had the lowest. Meanwhile, the expression level of the *CHH* gene in the muscle tissue of *E. sinensis* in the 22% starch group was significantly higher than that in the other two groups. Significant differences were also observed in the ecdysteroid receptor (*ECR*) gene among the three groups (*p* < 0.05), with the gene expression level gradually decreasing as the starch concentration increased. The 12% starch group had the highest *ECR* gene expression level, while the 32% starch group had the lowest. For the molt‐inhibiting hormone (*MIH*) gene, significant differences were found between the 32% starch group and the 12% and 22% starch groups (*p* < 0.05). The *MIH* gene expression level gradually increased with the increase in starch concentration, with the 32% starch group having the highest expression level, which was significantly higher than that in the 12% and 22% starch groups.

### 3.2. Effects of Different Dietary Starch Levels on Energy Metabolism of *E. sinensis*

The activities of major glycolytic enzymes in *E. sinensis* were significantly affected by dietary starch content. Table 4 shows the changes in glycolytic enzyme activities under different starch levels. As indicated in the table, the activities of HK, GDH, and PK did not show significant changes with the increase in dietary starch content. Glycogen content exhibited a fluctuating trend initially with the elevation of starch content, and the 12% starch group was significantly different from the other four groups (*p* < 0.05). The activity of LDH first increased and then decreased as starch content increased, with the highest activity observed at the 27% starch level, which was significantly higher than that in the other four groups (*p* < 0.05).

**Table 4:** Glycolytic enzymes in the hepatopancreas of *Eriocheir sinensis* fed diets with different starch levels (U/mgprot).

Group	12%	17%	22%	27%	32%
Glycogen	3.26 ± 0.17^a^	2.70 ± 0.02^b^	2.80 ± 0.06^b^	2.63 ± 0.26^b^	2.76 ± 0.07^b^
HK	55.52 ± 3.05	62.96 ± 8.27	68.64 ± 1.02	65.35 ± 3.77	58.33 ± 3.73
GDH	3.17 ± 0.20	3.49 ± 0.44	3.87 ± 0.37	3.72 ± 0.62	3.42 ± 0.13
LDH	14.82 ± 0.91^c^	16.66 ± 1.97^c^	22.35 ± 2.38^b^	29.38 ± 1.10^a^	22.79 ± 1.50^b^
PK	141.87 ± 2.66	143.90 ± 5.24	167.17 ± 3.01	166.76 ± 17.28	135.85 ± 14.66

*Note:* Letters superscripted to values in the same column indicate significant differences among values (*p* < 0.05).

As shown in Figure 2, the expression level of the PK gene in the hepatopancreas of *E. sinensis* first increased and then decreased, with the highest expression observed in the 22% starch group. Significant differences were found between the 22% group and the 12% and 32% groups (*p* < 0.05). The expression levels of the *HK* and *GLUT1* genes gradually increased with the elevation of starch concentration, with the highest expression in the 32% starch group, which was significantly higher than that in the 12% and 22% groups (*p* < 0.05).

**Figure 2:**
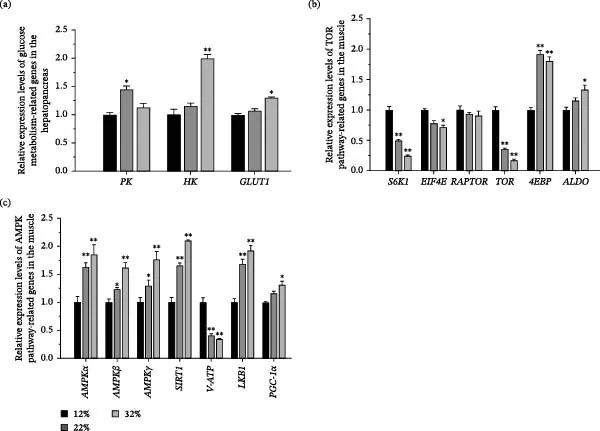
Effects of dietary starch levels on the relative expression of energy metabolism‐related genes in *Eriocheir sinensis*. Subpart (a) shows the expression of PK, HK, and GLUT1 in the hepatopancreas. Subpart (b) shows the expression of S6K1, EIF4E, RAPTOR, TOR, 4EBP, and ALDO in muscle. Subpart (c) shows the expression of AMPKα, AMPKβ, AMPKγ, SIRT1, V‐ATP, LKB1, and PGC‐1α in muscle. Relative expression was calculated using the $2^{-\Delta \Delta C_{t}}$ method, with β‐actin as the reference gene. Data are presented as mean ± SE.  ^∗^ indicates *p* < 0.05, and  ^∗∗^ indicates *p* < 0.01.

When fed diets with different starch contents, the relative expression levels of energy metabolism‐related genes in *E. sinensis* varied. For the *TOR* signaling pathway, the expression levels of *S6K1*, *EIF4E*, *RAPTOR*, and *TOR* genes gradually decreased with the increase in dietary starch content. In contrast, the expression level of the *4EBP* gene first increased and then decreased, with the highest expression in the 22% starch group, which was significantly different from the 12% and 32% groups (*p* < 0.05).

For the AMPK signaling pathway, the expression levels of *AMPKα*, *AMPKβ*, *AMPKγ*, *SIRT1*, *LKB1*, and *PGC-1α* genes increased with the increase in dietary starch content. The expression levels in the 12% and 22% groups were significantly lower than those in the 32% group (*p* < 0.05). However, the expression level of the *V-ATP* gene gradually decreased as the starch content increased.

### 3.3. Effects of Different Dietary Starch Levels on Energy Metabolites of *E. sinensis*

As shown in Table 5, among the 26 detected metabolites, the levels of 16 metabolites, including FMN, cyclic AMP (cAMP), and NAD, were the highest in the ED2 group. Among them, FMN showed significant differences among all groups (*p* < 0.05), and its level in the 22% starch group was highly significantly different from that in the 32% starch group and the other three groups (*p* < 0.01); the level of NADP in the 22% starch group was significantly different from that in the 32% and 12% starch groups (*p* < 0.05); the level of AMP in the 22% starch group was significantly different from that in the ED1 group (*p* < 0.05). In addition, under the experimental conditions, the levels of cAMP, NAD, oxaloacetic acid, NADPH, guanosine monophosphate (GMP), guanosine diphosphate (GDP), and other substances varied among groups, but the differences were not statistically significant (*p* > 0.05).

**Table 5:** Analysis of energy metabolite levels in the muscle of *Eriocheir sinensis* under different dietary starch levels.

Component name	Analyte mass range		Analyte retention time (min)	Expression of 32% group	Expression of 22% group	Expression of 12% group	Fold change (32% group/22% group)	Fold change (32% group/12% group)	Fold change (22% group/12% group)	*p*‐Value (32% group/22% group/12% group)
FMN	455.1/97.0	5.17		2.39E + 04	8.33E + 04	5.07E + 04	3.49 ^∗∗^	2.12 ^∗^	0.61 ^∗^	0.00 ^∗∗^
cAMP	328.0/134.0	2.02		2.02E + 04	8.15E + 04	3.57E + 04	4.04	1.77	0.44	0.07
NAD	662.1/540.0	6.42		1.52E + 05	4.31E + 05	2.89E + 05	2.84	1.90	0.67	0.24
L‐malic acid	133.0/115.1	5.28		2.71E + 08	1.79E + 08	2.31E + 08	0.66	0.85	1.29	0.24
NADP	742.1/620.0	10.24		2.50E + 05	6.33E + 05	4.24E + 05	2.53 ^∗^	1.70	0.67 ^∗^	0.27
Lactate	89.2/43.0	1.86		4.98E + 06	6.52E + 06	5.24E + 06	1.31	1.05	0.80	0.31
AMP	346.1/79.0	6.24		3.70E + 07	9.25E + 07	4.98E + 07	2.50 ^∗^	1.35	0.54	0.32
Fumaric acid	115.1/71.1	4.34		5.06E + 06	1.67E + 06	2.64E + 06	0.33	0.52	1.58	0.32
D‐3‐phosphoglycerate	185.0/97.0	8.03		3.07E + 05	5.21E + 05	1.14E + 06	1.70	3.72	2.19	0.33
GDP	442.0/79.0	9.32		5.64E + 04	1.14E + 05	8.07E + 04	2.01	1.43	0.71	0.34
Oxaloacetic acid	131.1/87.1	4.68		2.51E + 05	3.34E + 05	3.04E + 05	1.33	1.21	0.91	0.37
GMP	362.1/79.1	7.83		6.23E + 06	1.18E + 07	9.14E + 06	1.89	1.47	0.78	0.39
NADPH	744.1/408.0	10.24		3.49E + 03	6.73E + 03	2.68E + 03	1.93	0.77	0.40	0.42
PEP	167.1/79.0	7.74		4.59E + 04	6.57E + 04	1.12E + 05	1.43	2.44	1.70	0.43
Succinic acid	117.1/73.0	4.82		1.00E + 07	2.18E + 06	3.36E + 06	0.22	0.34	1.54	0.46
NADH	664.1/408.0	6.41		2.94E + 04	4.54E + 04	4.42E + 04	1.54	1.50	0.97	0.46
Citric acid	191.1/87.1	8.12		2.11E + 07	2.49E + 07	2.43E + 07	1.18	1.15	0.98	0.47
β‐D‐fructose‐6‐phosphate	259.1/97.0	7.40		1.08E + 07	1.97E + 07	3.54E + 07	1.83	3.28	1.79	0.47
ADP	426.0/79.0	7.62		2.76E + 07	4.44E + 07	3.22E + 07	1.61	1.17	0.72	0.50
D‐glucose‐6‐phosphate	259.0/97.0	8.36		1.23E + 07	2.11E + 07	4.55E + 07	1.72	3.71	2.15	0.52
ATP	506.0/159.0	8.99		1.79E + 05	2.38E + 05	2.68E + 05	1.33	1.50	1.13	0.52
Isocitric acid	191.0/73.0	7.93		1.10E + 05	1.71E + 05	1.32E + 05	1.55	1.19	0.77	0.54
Pyruvic acid	87.1/43.1	1.39		6.22E + 05	7.29E + 05	6.67E + 05	1.17	1.07	0.92	0.57
TPP	423.0/302.0	9.00		2.43E + 04	2.86E + 04	2.96E + 04	1.18	1.22	1.03	0.66
α‐Ketoglutaric acid	145.2/101.1	4.05		3.41E + 07	3.56E + 07	4.50E + 07	1.04	1.32	1.26	0.71
cis‐Aconitic acid	173.1/85.1	7.06		5.21E + 05	7.11E + 05	7.07E + 05	1.36	1.36	0.99	0.74

^∗^Indicates a significant difference (*p* < 0.05).

^∗∗^Indicates a highly significant difference (*p* < 0.01).

As shown in Figure 3a, among the 26 detected metabolites, 16 metabolites (including FMN, cAMP, and NAD) exhibited a trend of first increasing and then decreasing with the gradual elevation of dietary starch content. Among these, FMN, NADP, and AMP reached the highest levels in the 22% starch group, showing significant differences (*p* < 0.05). Four metabolites (including L‐malic acid and GDP) showed a trend of first decreasing and then increasing with the increase in starch content, while six metabolites (including succinic acid and ATP) showed a decreasing trend as starch content gradually increased. To more intuitively display the changes in metabolite levels, boxplot analysis was performed using normalized areas (Figure 3a,b).

**Figure 3:**
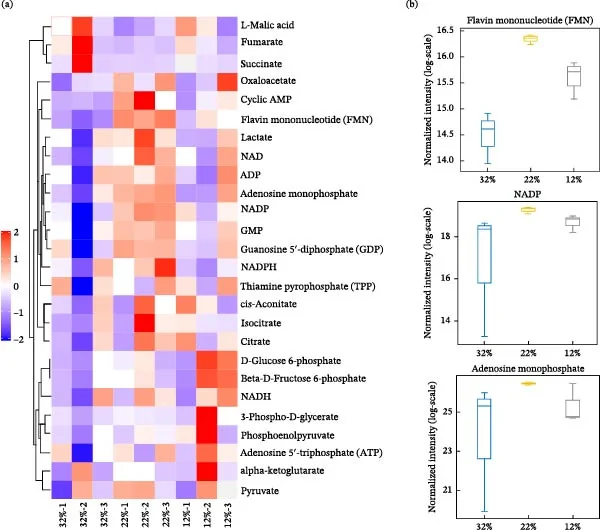
Effects of different dietary starch levels on energy metabolites in the muscle of *Eriocheir sinensis* (*X*‐axis: dietary starch level groups; *Y*‐axis: energy metabolites. (a) Heatmap of energy metabolites in the muscle of *Eriocheir sinensis*. (b) Targeted energy metabolites including flavin mononucleotide (FMN), nicotinamide adenine dinucleotide phosphate (NADP), and adenosine monophosphate (AMP). The *x*‐axis represents the 32%, 22%, and 12% starch groups in sequence; the *Y*‐axis: normalized intensity).

## 4. Discussion

### 4.1. Effects of Different Dietary Starch Levels on the Growth of *E. sinensis*

Starch serves as a primary energy source for organismal growth, reproduction, and metabolism, playing a crucial role in aquaculture [18]. Dietary starch content significantly influences the larval development of *E. sinensis*. Previous research has shown that zoea larvae of *E. sinensis* achieve the highest weight gain rate when fed diets with a starch content ranging from 18% to 26% [19], indicating that an appropriate starch concentration promotes the growth of *E. sinensis*. The results of the present study demonstrated that when dietary starch contents were 12%, 17%, 22%, 27%, and 32%, the growth indices of *E. sinensis* first increased and then decreased with the gradual elevation of starch content. Among these, the 22% starch group showed the best overall growth performance.

The MIH, ECR, and CHH genes are key regulatory genes involved in the molting and growth of *E. sinensis*, exerting complex regulatory effects on the growth of *E. sinensis* during its developmental process [20]. The inhibition of molting by the *MIH* gene and the promotion of molting by the *ECR* gene jointly regulate the molting cycle and thus influence growth, supported by CHH‐mediated metabolism [21]. In the present study, the expression differences of these growth‐related genes under different dietary starch levels were analyzed. The results showed that there were significant differences in *CHH* gene expression (*p* < 0.05), with the highest expression in the 22% starch group and the lowest in the 12% starch group. With the increase in dietary starch content, the *MIH* gene expression increased gradually, while the *ECR* gene expression decreased. These findings indicate that elevated starch content inhibits the molting of *E. sinensis*. The high *CHH* expression observed in the 22% starch group further suggests an association between energy metabolism and growth.

Previous studies have demonstrated that when the expression level of the *CHH* gene increases, the expression level of the *MIH* gene also rises within a certain range, and the data from the present study confirm that the elevation of *CHH* gene expression within a specific range can indeed promote the expression of the *MIH* gene [22].

### 4.2. Effects of Different Starch Levels on Energy Metabolism of *E. sinensis*

Amylase activity is widely recognized as an important indicator for evaluating the comprehensive digestion, absorption, and utilization of major nutrients in the diet of shrimp and crabs [23]. In the present study, the activities of HK, GDH, and PK did not change significantly with the increase in dietary starch content, indicating stable cellular energy supply within the tested starch concentration range. In contrast, LDH activity first increased and then decreased, reaching a peak in the 27% starch group, suggesting that appropriate starch intake can enhance the anaerobic metabolic efficiency and antioxidant capacity of *E. sinensis*. Previous studies have shown that excessive starch may cause metabolic disorders in aquatic animals, leading to oxidative stress or increased metabolic burden [24]. This phenomenon is consistent with the trend in glycogen content in the present study. As an important energy storage substance in glucose metabolism, glycogen content first increased and then decreased, with the highest level observed at 22% starch content. This indicates that there is a certain threshold for the storage and utilization of starch by *E. sinensis*, and excessively high starch content in the diet may interfere with glucose metabolism, thereby inhibiting glycogen synthesis and decomposition. Given the central role of the hepatopancreas in digestion, nutrient absorption, glycolipid metabolism, and enzyme synthesis and the role of muscle in movement‐ and growth‐related energy utilization, these two tissues jointly reflect metabolic regulation and energy allocation in crustaceans [25, 26].

The AMPK and TOR signaling pathways are important energy‐sensing pathways in animal tissue cells, playing a crucial role in maintaining cellular metabolism [27]. When cellular energy levels are low, the AMPK pathway participates in catabolic or anabolic metabolism to achieve cellular energy balance [28]. In the present study, the expression levels of key genes in the AMPK pathway showed an upward trend with increasing starch content, indicating that the AMPK pathway is activated when dietary starch levels increase, promoting energy storage and utilization in cells. Regarding the *V-ATP* gene, its expression level decreased with increasing starch content, suggesting that when dietary starch levels are low, the *V-ATP* gene functions in cells to provide energy and maintain normal physiological metabolism; whereas when dietary starch levels are high, the body has sufficient energy, and the *V-ATP* gene maintains cellular metabolic balance through regulation.

The TOR signaling pathway is a key regulatory pathway for growth and metabolic homeostasis, and the *4EBP* gene is a critical regulator within the TOR pathway [29]. The highest *4EBP* gene expression was observed in the 22% starch group, indicating that at this starch concentration, protein synthesis is accelerated through the regulation of the *4EBP* gene. In contrast, the expression levels of *S6K1*, *EIF4E*, *RAPTOR*, and *TOR* genes decreased with increasing dietary starch content. These decreases may help prevent metabolic overload during protein synthesis. Collectively, the results suggest that the AMPK and TOR pathways exert synergistic effects in mediating the regulatory responses of *E. sinensis* to dietary starch supplementation.

Glucose metabolism refers to the process of carbohydrate catabolism and anabolism, which maintains animal blood glucose homeostasis through glycolysis and gluconeogenesis [30]. *PK* regulates energy metabolism via glycolysis, supporting reproduction, stress responses, and growth and development [31]. In the present study, *PK* gene expression first increased and then decreased, peaking at a dietary starch content of 22%. This indicates that moderate starch intake can significantly activate glycolysis, enhance *PK* gene expression efficiency, and thereby support energy production and provide intermediates for other metabolic pathways. Additionally, *HK* and *GLUT1* gene expression levels continued to increase with increasing starch concentration, reaching the highest at 32% starch content. As a key enzyme in glycolysis, the upregulation of *HK* gene expression reflects enhanced glucose phosphorylation capacity in the organism [25]. Meanwhile, the increased expression of *GLUT1* indicates improved cellular glucose uptake efficiency [32]. It is speculated that the synergistic regulation of these two genes enhances glucose metabolism in *E. sinensis* under high‐starch conditions, thereby maintaining the dynamic balance of glucose metabolism [33].

### 4.3. Energy Metabolome Analysis of *E. sinensis* Under Different Dietary Starch Levels

Dietary starch levels achieve precise regulation of energy homeostasis by shaping the differential response patterns of energy metabolites in the muscle of *E. sinensis* [12]. FMN, a cofactor of flavoproteins, acts as a potent oxidant that participates in fatty acid β‐oxidation, amino acid synthesis, and other metabolic reactions, thereby influencing glucose and lipid metabolism in crabs [11]. In the present study, FMN content showed significant differences among groups (*p* < 0.05), with the highest level observed in the 22% starch group. This suggests that 22% dietary starch is conducive to FMN production, which in turn benefits the growth and metabolism of *E. sinensis*. NADP is a coenzyme involved in biosynthetic and redox reactions, whereas NADPH is its reduced form [34]. It plays a key role in protecting the organism from oxidative stress and maintaining energy metabolic transfer reactions in cells. In this experiment, NADP content in the 22% starch group was significantly higher than that in the 32% and 12% groups (*p* < 0.05), indicating that 22% dietary starch facilitates NADP accumulation, thereby effectively regulating energy metabolism in crabs. AMP, a nucleotide found in RNA, participates in various metabolic processes in crabs and serves as one of the main sources of energy supply. In the present study, AMP content in the 22% starch group was significantly higher than that in the 32% group (*p* < 0.05), suggesting that 22% dietary starch optimizes energy homeostasis in the organism. In contrast, high starch content (32%) may impair energy sensing and reduce energy conversion efficiency [35]. Regarding metabolites involved in the TCA cycle (e.g., NAD, NADPH, GMP, and oxaloacetic acid), they exhibited relatively large fold differences among groups, although the differences were not statistically significant (*p* > 0.05). This implies that under the optimal dietary starch level (22%), ATP production and metabolic activity are enhanced, reflecting potential metabolic advantages [36]. It is speculated that extending the experimental period appropriately may lead to significant differences in these metabolites.

### 4.4. The Relationship Between Growth and Energy Metabolism of *E. sinensis* Under Different Dietary Starch Levels

As an important carbohydrate source in aquatic feeds, dietary starch can indirectly influence growth performance by altering energy metabolism [37]. Growth in *E. sinensis* depends on the dynamic regulation of energy balance [26]. When the dietary starch level is within the appropriate range, starch is digested and decomposed into glucose, which can be efficiently converted into ATP through the glycolysis–TCA cycle, providing sufficient energy for growth‐related processes such as protein synthesis and cell proliferation. Meanwhile, intermediate products of glucose metabolism (e.g., glucose‐6‐phosphate and pyruvate) can also act as signaling molecules to optimize energy allocation by regulating pathways such as AMPK (energy sensor) and TOR (growth regulator), thereby influencing growth‐related physiological activities [38]. In the present study, the higher levels of FMN, NADP, AMP, and other energy‐related metabolites in the 22% starch group further suggested a more favorable status of redox balance, nucleotide metabolism, and energy transfer. These metabolic changes were consistent with the relatively better glycolytic response and AMPK/TOR–related gene expression patterns, indicating that moderate starch supply may coordinate energy production and growth‐related biosynthesis. Therefore, the growth advantage observed at 22% dietary starch may be associated with the coordinated regulation of glycolysis, muscle energy metabolite accumulation, and AMPK/TOR–mediated energy allocation [5, 11, 12]. If the starch level is too low, the crab’s body has to rely on fat or protein decomposition for energy supply, which not only increases metabolic costs but may also limit the expression of growth‐related genes and material synthesis due to energy shortage [3]. Conversely, excessively high starch levels are prone to inducing glucose metabolism disorders, leading to imbalanced glycolytic enzyme activities, insufficient accumulation of energy metabolites, and activation of the AMPK pathway to inhibit TOR signaling, ultimately impairing growth efficiency [39]. The trend that the growth performance of *E. sinensis* first increased and then decreased with increasing starch levels in this study is essentially an adaptive response of energy metabolism to starch supply: The appropriate starch level (22%) provides efficient and stable energy support for growth by positively regulating key nodes of energy metabolism.

## 5. Conclusion

This study systematically analyzed the response patterns of growth performance, activities of enzymes involved in energy metabolism, gene expression, and muscle energy metabolites in *E. sinensis*. The results showed that the growth performance of *E. sinensis* exhibited a trend of first increasing and then decreasing with the elevation of dietary starch levels, with the optimal growth achieved at a dietary starch level of 22%. In terms of energy metabolism, at the 22% dietary starch level, the activities of key glycolytic enzymes, the expression of related genes, and the expression of genes involved in the AMPK/TOR signaling pathways all exhibited characteristics consistent with growth. Meanwhile, the accumulation of energy metabolites in the muscle also supported the growth advantage of crabs in this group. For the first time, this study revealed the correlation mechanism between the growth of *E. sinensis* and dietary starch levels from the perspective of energy metabolism, confirming that 22% is the appropriate dietary starch addition level for *E. sinensis*. This finding can provide a scientific basis for the precision formula design of compound feeds for *E. sinensis* and offer a theoretical reference at the metabolic level for research on carbohydrate nutrition in crustaceans. From a practical perspective, ~22% dietary starch may serve as a reference level for juvenile *E. sinensis* feed formulation under similar culture conditions.

## Author Contributions

**Yanan Liu**: formal analysis, visualization, writing – review and editing. **Xiaojie Zhou**: investigation, supervision, writing – original draft. **Sudong Xia**, **Zhenyan Cheng, and Jinwei Gao**: investigation, supervision, validation, writing – review and editing. **Wenli Zhou and Dan Zhang**: conceptualization, investigation, writing – review and editing.

## Funding

The authors declare that financial support was received for the research, authorship, and publication of this article. This work was supported by the National Key Research and Development Program of China (Grant 2024YFD2401803), the Scientific Research Programme of Tianjin Municipal Education Commission (Key Project of Natural Science, Grant 2023ZD001), and the Tianjin Municipal Science and Technology Plan Project (Grant 25ZXBTSN00010).

## Disclosure

All claims expressed in this article are solely those of the authors and do not necessarily represent those of their affiliated organizations or those of the publisher, the editors, and the reviewers. Any product that may be evaluated in this article, or claim that may be made by its manufacturer, is not guaranteed or endorsed by the publisher.

## Ethics Statement

*Eriocheir sinensis* is not an endangered species. The legal requirements in the country for the care and use of animals have been followed. The Animal Ethics Committee’s scope of approval is limited to studies involving vertebrates (Laboratory Animal Act, Chapter 5, Article 25). No approval from a research ethics committee was required to accomplish the goals of this study because experimental work was conducted with the unregulated invertebrate *Eriocheir sinensis*.

## Conflicts of Interest

The authors declare no conflicts of interest.

## Data Availability

The original contributions presented in the study are included in the article. Further inquiries can be directed to the corresponding author.

## References

[1] Wang S. , Xu G. , and Zou J. , Soluble Non-Starch Polysaccharides in Fish Feed: Implications for Fish Metabolism, Fish Physiology and Biochemistry. (2024) 50, no. 1, 1–22, 10.1007/s10695-022-01131-y. 36219350

[2] Okon E. , Iyobhebhe M. , and Olatunji P. , et al.Feed Additives in Aquaculture: Benefits, Risks, and the Need for Robust Regulatory Frameworks, Fishes. (2025) 10, no. 9, 10.3390/fishes10090471.

[3] Singha K. P. , Sahu N. P. , Sardar P. , Shamna N. , and Kumar V. , A Strategic Roadmap for Carbohydrate Utilization in Crustaceans Feed, Reviews in Aquaculture. (2024) 16, no. 2, 674–705, 10.1111/raq.12861.

[4] Karim A. , Naila B. , and Khwaja S. , et al.Evaluation of Different Starch Binders on Physical Quality of Fish Feed Pellets, Brazilian Journal of Biology. (2024) 84, 10.1590/1519-6984.256242, e256242. 35195178

[5] Li T. , Li E. , and Suo Y. , et al.Energy Metabolism and Metabolomics Response of Pacific White Shrimp *Litopenaeus vannamei* to Sulfide Toxicity, Aquatic Toxicology. (2017) 183, 28–37, 10.1016/j.aquatox.2016.12.010. 27988416

[6] Yuan Y. , Jin M. , and Fang F. , et al.New Insight Into the Molting and Growth in Crustaceans: Regulation of Energy Homeostasis Through the Lipid Nutrition, Frontiers in Marine Science. (2022) 9, 10.3389/fmars.2022.914590, 914590.

[7] Wu K. , Li Z. , and Hou D. , et al.Ace2 Mutation Disrupts Amino Acid Absorption, Impairs Growth, and Alters Microbiota Dynamics in Zebrafish, Communications Biology. (2025) 8, no. 1, 10.1038/s42003-025-08626-3. PMC12354748 40813900

[8] Li X. , Li X. , and Wu Z. , et al.Recent Advances in Nutritional Requirements and Metabolic Homeostasis Regulation of Animals Under Stress Conditions, Animals. (2025) 15, no. 23, 10.3390/ani15233412. PMC12691100 41375469

[9] Kulkarni P. P. , Regulation and Role of AMP-Activated Protein Kinase at the Cellular Level and Relevance to Diabetes Mellitus, Chronicle of Diabetes Research and Practice. (2022) 1, no. 1, 20–30, 10.4103/cdrp.cdrp_5_21.

[10] Li D. , Ding Y. , and Cheng L. , et al.Target of Rapamycin (TOR) Regulates the Response to Low Nitrogen Stress via Autophagy and Hormone Pathways in *Malus hupehensis* , Horticulture Research. (2022) 9, 10.1093/hr/uhac143, uhac143. PMC9437726 36072834

[11] Schnerwitzki D. and Vabulas R. M. , Dynamic Association of Flavin Cofactors to Regulate Flavoprotein Function, IUBMB Life. (2022) 74, no. 7, 645–654, 10.1002/iub.2591. 35015339

[12] Zuo R. , Wen B. , Jiang Y. , Huang S. , and Yi Q. , Growth, Biochemical Indices and Transcriptomic Profile of Chinese Mitten Crab (*Eriocheir sinensis*) Respond to Different Ratios of Dietary Carbohydrates to Lipids, Frontiers in Marine Science. (2023) 10, 10.3389/fmars.2023.1176976, 1176976.

[13] Xu F. , Xing X. , and Zhang K. , et al.Status and Prospects of Product Processing and Sustainable Utilization of Chinese Mitten Crab (*Eriocheir sinensis*), Heliyon. (2024) 10, no. 12, 10.1016/j.heliyon.2024.e32922, e32922. PMC11238004 38994063

[14] Long X. , Pan J. , and Wade N. M. , et al.Effects of Three Feeding Modes on the Metabolism, Antioxidant Capacity, and Metabolome of the Adult Male Chinese Mitten Crab *Eriocheir sinensis* , Aquaculture International. (2022) 30, no. 3, 1101–1119, 10.1007/s10499-021-00782-7.

[15] Zhao L. , Yang X. , and Cheng Y. , et al.Effect of Dietary Histamine Supplementation on Growth, Digestive Enzyme Activities and Morphology of Intestine and Hepatopancreas in the Chinese Mitten Crab *Eriocheir sinensis* , SpringerPlus. (2016) 5, no. 1, 10.1186/s40064-016-2105-9. PMC4851676 27190751

[16] Miao S. , Hu J. , and Wan W. , et al.Effects of Graded Levels of Starch on the Non-Specific Immune Responses, Antioxidant Capacities and Intestinal Health in Chinese Mitten Crab, *Eriocheir Inensis* , Fish & Shellfish Immunology. (2020) 104, 402–409, 10.1016/j.fsi.2020.06.035. 32562867

[17] Witting M. and Böcker S. , Current Status of Retention Time Prediction in Metabolite Identification, Journal of Separation Science. (2020) 43, no. 9-10, 1746–1754, 10.1002/jssc.202000060. 32144942

[18] Ma H.-J. , Mou M.-M. , Pu D.-C. , Lin S.-M. , Chen Y.-J. , and Luo L. , Effect of Dietary Starch Level on Growth, Metabolism Enzyme and Oxidative Status of Juvenile Largemouth Bass, *Micropterus salmoides* , Aquaculture. (2019) 498, 482–487, 10.1016/j.aquaculture.2018.07.039.

[19] Weng C.-Y. , Sun J.-Y. , and Chen Y.-C. , et al.New Insights Into Feeding Behavior and Digestive Physiology of the Chinese Mitten Crab (*Eriocheir sinensis*) Zoea 5 (Z5) and Megalopa (M), Aquaculture. (2025) 598, 10.1016/j.aquaculture.2024.742037.

[20] Li C. , Huang L. , and Zhang Y. , et al.Effects of Triazole Plant Growth Regulators on Molting Mechanism in Chinese Mitten Crab (*Eriocheir sinensis*), Fish & Shellfish Immunology. (2022) 131, 646–653, 10.1016/j.fsi.2022.10.059. 36330873

[21] Gismondi E. , Identification of Molt-Inhibiting Hormone and Ecdysteroid Receptor cDNA Sequences in *Gammarus pulex*, and Variations After Endocrine Disruptor exposures, Ecotoxicology and Environmental Safety. (2018) 17. 10.1016/j.ecoenv.2018.04.017 29656166

[22] Cervellione F. , Mcgurk C. , and Berger Eriksen T. , et al.Effect of Starvation and Refeeding on the Hepatopancreas of Whiteleg Shrimp *Penaeus Vannamei*(Boone)using Computer-Assisted Image Analysis, Journal of Fish Diseases. (2017) 40, no. 11, 1707–1715, 10.1111/jfd.12639. 28493487

[23] Rodin A. M. , Kuznetsova V. M. , and Krupnova Y. M. , et al.Activity of Energy and Carbohydrate Metabolism Enzymes in Rainbow Trout Fingerlings (oncorhynchus Mykiss Walb.) when Fed Two Types of Commercial Feed, Biology Bulletin. (2024) 51, no. 6, 1539–1548.

[24] Xie Y. , Shao X. , and Zhang P. , et al.High Starch Induces Hematological Variations, Metabolic Changes, Oxidative Stress, Inflammatory Responses, and Histopathological Lesions in Largemouth Bass (*Micropterus salmoides*), Metabolites. (2024) 14, no. 4, 10.3390/metabo14040236. PMC11051861 38668364

[25] Wang X. D. , Li E. C. , and Qin J. , et al.Growth, Body Composition, and Ammonia Tolerance of Juvenile White Shrimp *Litopenaeus vannamei* Fed Diets Containing Different Carbohydrate Levels at Low Salinity, Journal of Shellfish Research. (2014) 33, no. 2, 511–517, 10.2983/035.033.0220.

[26] Yu N. , Ding Q. , and Li E. , et al.Growth, Energy Metabolism and Transcriptomic Responses in Chinese Mitten Crab (*Eriocheir sinensis*) to Benzo [α] Pyrene (BaP) Toxicity, Aquatic Toxicology. (2018) 203, 150–158, 10.1016/j.aquatox.2018.08.014. 30138799

[27] Chen X. , Hou Y. , and Liao A. , et al.Integrated Analysis of Gut Microbiome and Adipose Transcriptome Reveals Beneficial Effects of Resistant Dextrin From Wheat Starch on Insulin Resistance in Kunming Mice, Biomolecules. (2024) 14, no. 2, 10.3390/biom14020186. PMC10886926 38397423

[28] Corsetti G. , Pasini E. , and Scarabelli M. T. , et al.Importance of Energy, Dietary Protein Sources, and Amino Acid Composition in the Regulation of Metabolism: An Indissoluble Dynamic Combination for Life, Nutrients. (2024) 16, no. 15, 2417–2417, 10.3390/nu16152417. PMC11313897 39125298

[29] Ming L. , Xin Z. , and Lefei J. , et al.Dietary Protein Regulates Ovarian Development Through TOR Pathway Mediated Protein Metabolism in Female *Litopenaeus vannamei* , Aquaculture Reports. (2023) 33, 101781.

[30] Salih K. J. , Sabir D. K. , and Abdoul H. J. , Glycolysis Regulation to Maintain Blood Glucose Homeostasis, Kurdistan Journal of Applied Research. (2022) 7, no. 1, 114–124.

[31] Dildar T. , Cui W. , and Dai S. , et al.Multi-Omics Insights Into Energy Metabolism Changes in Response to Salinity Adaptation in Mud Crab (*Scylla paramamosain*), International Journal of Biological Macromolecules. (2026) 337, no. Pt 2, 10.1016/j.ijbiomac.2025.149641, 149641. 41390036

[32] Tang R. , Zhong T. , and Fan L. , et al.Enhanced T Cell Glucose Uptake Is Associated With Progression of Beta-Cell Function in type 1 Diabetes, Frontiers in Immunology. (2022) 13, 10.3389/fimmu.2022.897047, 897047. PMC9168918 35677051

[33] Liu H. , Feng Y. , and Yang M. , et al.Starvation Induces Hepatopancreas Atrophy in Chinese Mitten Crab (*Eriocheir sinensis*) by Inhibiting Angiogenesis, BioMed Central Genomics. (2023) 24, no. 1, 10.1186/s12864-023-09620-x. PMC10571328 37828424

[34] Cheng C.-H. , Dong J.-D. , and Fan S.-G. , et al.The Role of NADPH Oxidase in the Mud Crab (*Scylla paramamosain*) in Response to *Vibrio parahaemolyticus* Infection, Comparative Biochemistry and Physiology Part C: Toxicology & Pharmacology. (2025) 292, 10.1016/j.cbpc.2025.110158, 110158. 39993587

[35] Sokolova I. M. , Energy-Limited Tolerance to Stress as a Conceptual Framework to Integrate the Effects of Multiple Stressors, Integrative & Comparative Biology. (2013) 4, 597–608. 10.1093/icb/ict028 23615362

[36] Wang S.-H. , Li C.-T. , and Shang M. , et al.Identification and Genetic Diversity Analysis of Chinese Mitten Crab(*Eriocheir sinensis*) in the Liao River Area, Journal of Northeast Agricultural University. (2018) 25, no. 2, 43–53.

[37] Feng Z. , Zhong Y. , and He G. , et al.Yeast Culture Improved the Growth Performance, Liver Function, Intestinal Barrier and Microbiota of Juvenile Largemouth Bass (*Micropterus salmoides*) Fed High-Starch Diet, Fish & Shellfish Immunology. (2022) 120, 706–715, 10.1016/j.fsi.2021.12.034. 34954371

[38] Jin M. , Luo J. , and Zhu T. , et al.Examination of Role of the AMP-Activated Protein Kinase (Ampk) Signaling Pathway During Low Salinity Adaptation in the Mud Crab, *Scylla paramamosain*, With Reference to Glucolipid Metabolism, Aquaculture. (2024) 593, 10.1016/j.aquaculture.2024.741329, 741329.

[39] Bian F. , Jiang H. , and Man M. , et al.Dietary Gossypol Suppressed Postprandial TOR Signaling and Elevated ER Stress Pathways in Turbot (*Scophthalmus maximus* L.), American Journal of Physiology-Endocrinology and Metabolism. (2017) 312, no. 1, E37–E47, 10.1152/ajpendo.00285.2016. 27894064

